# Structural and Topographic Dynamics of Pulmonary Histopathology and Local Cytokine Profiles in *Paracoccidioides brasiliensis* Conidia-Infected Mice

**DOI:** 10.1371/journal.pntd.0001232

**Published:** 2011-07-12

**Authors:** Damaris Lopera, Tonny W. Naranjo, Oswaldo G. Cruz, Angela Restrepo, Luz Elena Cano, Henrique Leonel Lenzi

**Affiliations:** 1 Medical and Experimental Mycology Group, Corporación para Investigaciones Biológicas, Medellín, Colombia; 2 School of Health Sciences, Universidad Pontificia Bolivarina, Medellín, Colombia; 3 Programa de Computação Científica, Fundação Oswaldo Cruz, Rio de Janeiro, Brazil; 4 Microbiology School, Universidad de Antioquia, Medellín, Colombia; 5 Laboratory of Pathology, Instituto Oswaldo Cruz, Fundação Oswaldo Cruz, Rio de Janeiro, Brazil; Hospital Universitário, Brazil

## Abstract

**Background:**

Paracoccidioidomycosis (PCM), an endemic systemic mycosis caused by the fungus *Paracoccidioides brasiliensis (Pb)*, usually results in severe lung damage in patients.

**Methods and Findings:**

Considering the difficulties to sequentially study the infection in humans, this work was done in mice inoculated intranasally with infective *Pb-*conidia. Lungs of control and *Pb*-infected mice were studied after 2-hours, 4, 8, 12 and 16-weeks post-infection (p.i) in order to define histopathologic patterns of pulmonary lesions, multiplex-cytokine profiles and their dynamics during the course of this mycosis. Besides the nodular/granulomatous lesions previously informed, results revealed additional non-formerly described lung abnormalities, such as periarterial sheath inflammation and pseudotumoral masses. The following chronologic stages occurring during the course of the experimental infection were defined: Stage one (2-hours p.i): mild septal infiltration composed by neutrophils and macrophages accompanied by an intense “cytokine burst” represented by significant increases in IL-1α, IL-1β, IL-4, IL-5, IL-6, IL-10, IL12p70, IL-13, IL-17, Eotaxin, G-CSF, MCP1, MIP1α, GM-CSF, IFN-γ, MIP1β and TNFα levels. Stage two (4-weeks p.i): presence of nodules, evidence of incipient periarterial- and intense but disperse parenchymal- inflammation, abnormalities that continued to be accompanied by hyper-secretion of those cytokines and chemokines mentioned in the first stage of infection. Stages three and four (8 and 12-weeks p.i.): fungal proliferation, inflammation and collagenesis reached their highest intensity with particular involvement of the periarterial space. Paradoxically, lung cytokines and chemokines were down-regulated with significant decreases in IL-2,IL-3,IL-5,IL-9,IL-13,IL-15,GM-CSF,IFN-γ,MIP1β and TNFα. Stage five (16-weeks p.i.): inflammation decreased becoming limited to the pseudotumoral masses and was accompanied by a “silent” cytokine response, except for PDGF, MIG, RANTES and IL12p40 which remained up-regulated for the duration of the experiment.

**Conclusions:**

Results of this study identified both classic and novel patterns corresponding to histopathologic and immunologic responses occurring during the course of experimental PCM.

## Author Summary

Paracoccidioidomycosis (PCM), an endemic fungal infection of pulmonary origin resulting in severe disseminated disease, occurs in rural areas of most South American countries and presents several clinical forms. The infection is acquired by inhalation of specific fungal propagules, called conidia. Considering the difficulties encountered when studying the infection in humans, this work was done in mice infected by inhalation of infective fungal conidia thus mimicking the human natural infection. The lungs of mice were sequentially studied by histopathological and multiplex cytokine methods from 2 h to 16 weeks after infection to verify the course of the disease. The mycosis presented different morphologic aspects during the course of time, affecting several pulmonary compartments. Otherwise and based on the analysis of 30 cytokines, the immune response also showed heterogeneous responses, which were up or down regulated depending on the time of infection. By recognizing the different stages that correspond to the evolution of pulmonary lesions, the severity (benign, chronic or fibrotic) of the disease could be predicted and the probable prognosis of the illness be inferred.

## Introduction

The chronic adult form of PCM progresses slowly with minor symptoms and may take months to years to become clinically manifested; consequently, when the patients are finally diagnosed and receive medical treatment, pulmonary lesions are already well established, presenting different stages of development [1]–[3].

The primary infection takes place in the lungs where the fungus may persist inactive for years. Tuder *et al.* (1985), in twelve patients with chronic PCM, described five main pathologic aspects: (a) pneumonic reaction; (b) early granulomatous formation; (c) mature and healed granulomas; (d) mixed pattern (early and mature granuloma in the same pulmonary area); (e) pulmonary fibrosis, with the latter being present in all cases [4]. In addition, in chronic human PCM, the lesions may be either diffuse or circumscribed [3]. Human studies, however, do not allow the histopathologic sequential analyses required to build up the sequence of events. Therefore, several experimental models have been applied to compensate this limitation, aiming to better define the pathogenic mechanisms in PCM [5]–[11]. Despite important differences in the experimental designs due to infective forms (conidia, yeasts) and fungal isolates used, inoculum concentration, route of inoculation and host genetic background, overall results have contributed to determine some stages of the inflammatory dynamics, delineate cellular and extracellular matrix composition of the granulomas and related aspects [5]–[11]. Nonetheless, a detailed description of the topographic location of the lesions in the lung compartments and the characteristics of other inflammatory patterns, besides the granulomatous reaction, had been incomplete and superficially referred to in the literature. Additionally, the immunological studies reported have been focused on specific cell populations or tested a restricted number of cytokines produced *in vivo* or *in vitro* [12]–[14]. Therefore, the aim of this study was to sequentially determine histopathologic patterns of lesions and multiplex-determined cytokine profiles in an experimental model of PCM.

A deeper knowledge of the pattern of lesions and cytokines profiles induced by the fungus and their changes according to the time of infection could contribute to prognosis prediction.

## Materials and Methods

### Ethics statement

All animals were handled according to the national (Law 84 of 1989, Res No. 8430 of 1993) and international (Council of European Communities and Canadian Council of Animal Care, 1998) guidelines for animal research and the experimental protocols were approved by Corporación para Investigaciones Biológicas (CIB) research ethics committee.

### Animals

BALB/c mice were originally obtained from Taconic Farms, Inc., Quality Laboratory Animals and Services for Research, New York, USA, with the breeding colony being expanded at the (CIB), Medellin, Colombia. Male mice, 6–7 weeks old and approximately 20 g in weight were used in this study. Mice were divided into 2 groups: non-infected control mice (*n = 50*) and infected mice (*n = 50*).

### Fungus

*Paracoccidioides brasiliensis* isolate ATCC-60855 from a Colombian patient registered at the American Type Culture Collection (Rockville, MD), and known to produce abundant conidia was used in all experiments [15]. The techniques applied to grow the mycelial forms with subsequent collection and dislodgement of conidia had been previously reported [15]–[17].

### Experimental infection

Mice were anesthetized by the intramuscular injection of a solution containing -ketamin hydrochloride (Park, Davis & Company, Berlin, Germany; 100 mg/kg) and xylazine (Bayer, Brazil 10 mg/kg) [18]. When deep anesthesia was obtained, 3×10^6^ *Pb* viable conidia (in 0.06 ml of the inoculum) were intranasally instilled. Control mice received an intranasal inoculum of 0.06 ml of PBS.

### Histopathologic analysis

Ten animals (5 control and 5 *Pb*-infected mice) were euthanized by thiopental overdose (Sandoz GmbH. Kundl Austria; 1 ml at 2.5%, i.p) at 2 hours and 4, 8, 12 and 16 weeks post-inoculation in accordance to animal ethical practices. Lungs of mice were intracardiacally perfused with 10% neutral formalin in phosphate-buffered saline, removed and fixed in the same solution for at least 48 h. Fixed lungs were embedded in paraffin and coronal sections (5 µm) were stained by the following methods: hematoxylin-eosin (HE); Grocott's methenamine-silver nitrate technique (Grocott) for identification of fungi; Lennert's Giemsa for cellular characterization [19]; Sirius red pH 10.2 for eosinophils [20], [21]; Weigert's resorcin-fuchsin for elastic fibers; Masson' trichrome; PAS and PAS-Alcian Blue pH 2.5 and 1.0 for proteoglycans and neutral glycoproteins; Gomori's silver reticulin for reticulin fibers and picrosirius with fast green (PIFG), for identification of collagen I and III [22]. Slides were automatically scanned by ScanScope® CS (Aperio, USA) and also analyzed with Axio Observer.Z1 (Zeiss), coupled with digital camera AxioCam HRc and software AxioVision 4.7.2 (Zeiss). Representative specimens were also stained with Phosphomolibdic acid-Picrosirius red (PMA-PS) [23] for interstitial collagen and analyzed by confocal microscopy (LSM 510 Meta-Zeiss).

### Image analysis

Location, shape, size, frequency, cellular composition, fungal budding patterns, and collagen deposits features were evaluated for each inflammatory pattern using scanned slides images and the free ImageScope software (http://www.aperio.com/download-imagescope-viewer.asp). Tissue sections were examined and evaluated blindly by a pathologist.

The total inflammatory area was measured by using one panoramic image of both lungs per mouse. Regions of interest (ROIs), correspondent to the inflammatory regions, were manually drawn and measured. The percentage of pulmonary area with inflammatory reaction was calculated dividing the sum of total ROIs by the total area of lung (excluding the air space).

The Aperio positive pixel count algorithm was used to quantify the amount of a specific stain present in a scan slide image. Red pixels in the PIFG stained slides were measured to quantify collagen, and brown/black pixels in the Grocott's stained slides to quantify yeast cells.

Cellular composition was evaluated using a semi-quantitative approach. A score of − to +++ was given according to the degree of infiltration with (+++) for intense, (++) for moderate, (+) for slight, (±) for very slight and (−) for no reaction [24].

### Immunohistology

Rabbit immunoglobulin to human fibronectin (DAKO, Cat # A245), known to react with fibronectin-covered *Pb* yeasts [25], was used to better observe the yeasts into a “perifungal space”. Indirect immunofluorescence was done on paraffin lung sections as described [25]. All slides were counterstained with Evans blue at a dilution of 1/10.000 and mounted in buffered glycerol containing p-phenylenediamine and analyzed by confocal microscopy (LSM-510-META, Zeiss).

### Cytokines, chemokines, and growth factors detection by multiplex microbead immunoassay

Lungs of five animals per group were individually homogenized in tissue grinder (Tissue tearor, model 985-370, Biospec Products) with a cocktail solution of protease inhibitors (Pepsin 0.1 uM, Leupeptin 0.1 uM, Phenylmethyl sulfonide fluoride 1 mM, N-tosil-L-Phenilalamine choromethyl ketone 0.2 mM, (a)-p-methyl L lisyne choromethyl ketone 0.1 mM from Sigma chemical, plus ethylene diamine tetra-acetic acid EDTA 1 nM from Merck Germany). Homogenized lungs supernatant were collected by centrifugation at 3000 rpm for 15 min at 4°C, aliquoted and stored at −70°C until the analysis day.

All homogenized lung supernatants were normalized to 1 mg/ml of protein. Then, a magnetic bead-based multiplex assay, containing fluorescent dyed microspheres conjugated with a monoclonal antibody specific for a target protein, was used for cytokines, chemokines, and growth factors measurement according to the manufacturer's instructions (Bio-Plex pro-mouse cytokine 23-plex assay and group II customized 8-plex assay; Bio-Rad Inc., Hercules, CA, USA). Molecules measured were: IL-1-α ,IL-1β, IL-2, IL-3, IL-4, IL-5, IL-6, IL-9, IL-10, IL-12 (p40), IL-12 (p70), IL-13, IL-15, IL-17, Eotaxin, granulocyte colony stimulating factor (G-CSF), granulocyte-monocyte colony stimulating factor (GM-CSF), IFN-γ, KC/CXCL1, monocyte chemoattractive protein (MCP-1/CCL2), macrophage inflammatory protein-1 alpha and beta (MIP-1α/CCL3 and MIP-1β/CCL4), RANTES (CCL5), TNF-α, leukocyte inhibitory factor (LIF), vascular endothelial growth factor (VEGF), platelet-derived growth factor (PDGF), basic fibroblast growth factor (FGFb), monokine induced by IFN-γ (MIG/CXCL9) and macrophage inflammatory protein-2 (MIP-2/CXCL2).

Cytokine levels were determined using a multiplex array reader from Luminex™ Instrumentation System (Bio-Plex Workstation from Bio-Rad Laboratories). The analyte concentration was calculated using software provided by the manufacturer (Bio-Plex Manager Software) and expressed as pg.

### Statistical analysis

Histopathology results were obtained from five biological replicates at each time point. Multiplex cytokine bioplex assays were analyzed in five additional animals at each time and run in duplicates.

Cytokine data analysis was performed through the use of R language (R Development Core Team, 2010). R: A language and environment for statistical computing. (R Foundation for Statistical Computing, Vienna, Austria. ISBN 3-900051-07-0, URL http://www.R-project.org). Cytokine data were initially analyzed by exploratory data analysis (EDA). The variables were individually plotted with 40% of smoothing by lowess (local regression), aiming to define the behavior of each variable during the infection. *Pb*-infected and control groups were compared by non-parametric Kruskal-Wallis test with Dunn's multiple comparisons post-test (Prism 5.0 software, Graph Pad, USA) dividing the groups up to four and after eight weeks of infection. *p* values less than 0.05 were considered statistically significant; *p* values less than 0.01 were considered statistically highly significant.

## Results

### Histopathological aspects

**Figure 1:**
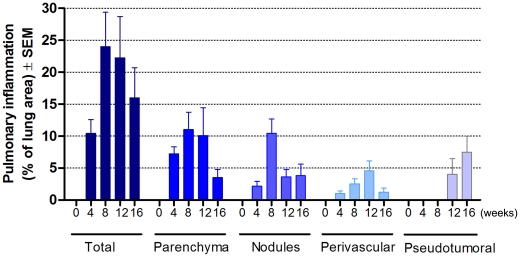
Quantification of different types of pulmonary inflammation observed during the course of chronic experimental PCM. The bar charts show the percentage of pulmonary area occupied by each type of histopathologic pattern of lesions at 4, 8, 12 and 16 weeks post *Pb*-infection in mice.

None of the animal receiving PBS exhibited histopathologic alterations except transient and slight septal infiltration of neutrophils with some eosinophils at 2 h post-inoculations. In comparison, the lungs of BALB/c mice infected with *Pb* conidia exhibited, from the 4 weeks onwards, pulmonary lesions with three main histopathologic patterns: the classical and commonly reported nodular or granulomatous pattern, followed by inflammatory peri-arterial sheaths and pseudotumoral masses. A quantitative evaluation of those inflammatory infiltrates is illustrated in the **Figure 1**.

#### Nodular pattern

**Figure 2:**
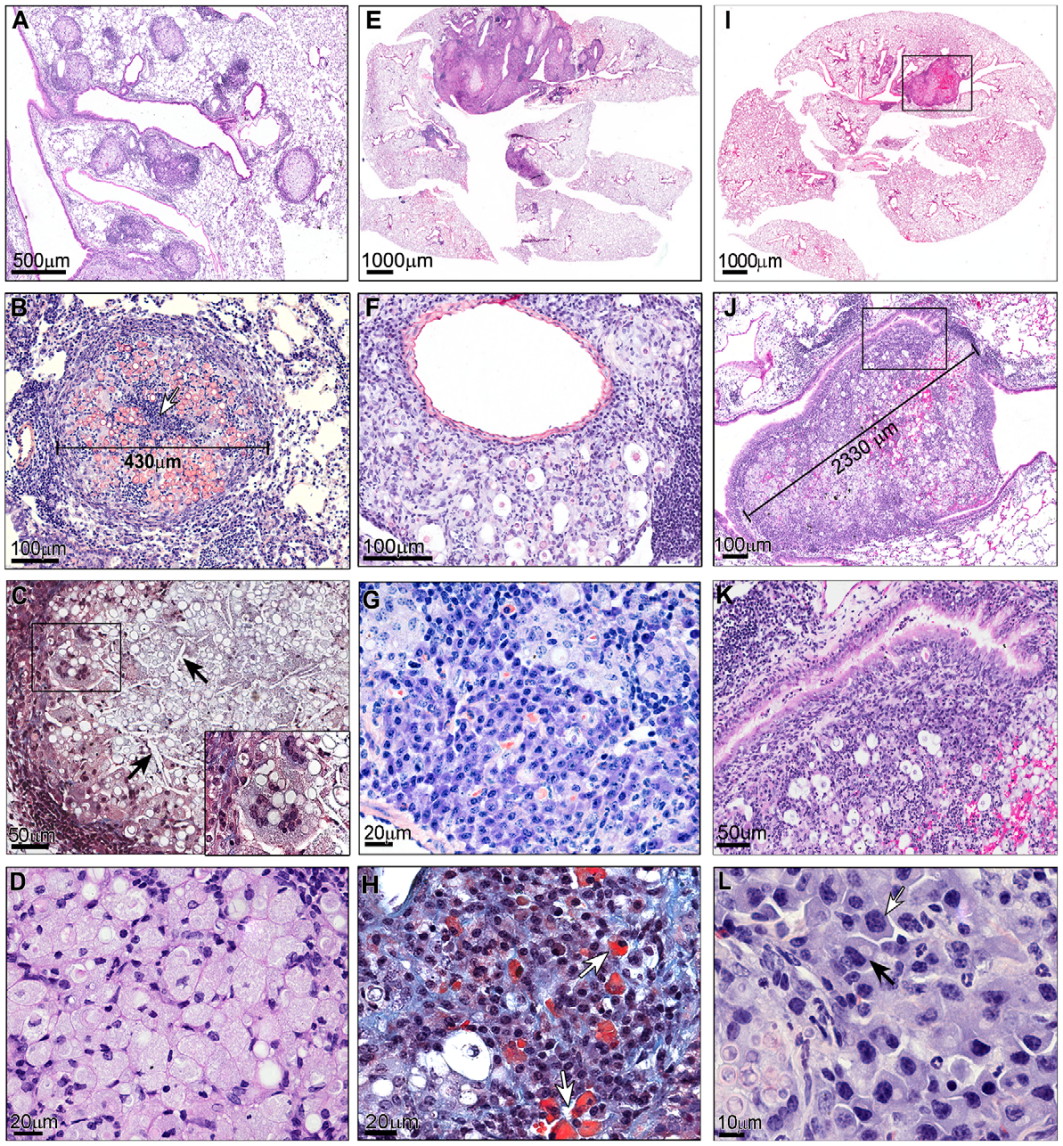
Main histopathologic patterns observed in the lung of *Pb* conidia- infected mice. Nodules (A to D), periarterial sheath inflammation (E to H) and pseudotummoral mass (I to L). (A) Multiple nodules in a panoramic view (3.5×), H&E. (B) Typical granuloma structure with intermixed PMN (arrow), Sirius red pH 10.2, 10×. (C) Granuloma with atheromatous appearance showing multiple cholesterol crystals (arrows) and Touton giant cells (insert, 100×), Masson's trichrome stain, 20×. (D) Xantomatous macrophages with foamy cytoplasm in the central zone of granuloma (63×), H&E. (E) Panoramic view of a complete coronal lung section (12^th^ wk p.i) showing extensive inflammatory consolidation in the left lung with multiple periarterial lesions (0.4×), H&E. (F) Periarterial sheath inflammation showing also several fungi enclosed in perifungal spaces (40×), Sirius red pH 10.2. (G) Large number of plasmocytes and small macrophage aggregates (63×), Lennert's Giemsa. (H) Mott cells containing Russell's bodies (cells in red) (63×), Masson's trichrome stain. (I) Panoramic view of a coronal lung section (16^th^ wk p.i) showing a pseudotumoral mass in the left lung obstructing the bronchial lumen (0.4×), H&E. (J) Detail of pseudotumoral mass presented in figure I showing its large dimension (20×), Sirius red pH 10.2, (K) Bronchial obstruction remarked in F, H&E, 20×. (L) Focus of plasmocytogenesis expressed by plasmoblasts and pro-plasmocytes (arrow) in the periphery of a pseudotumoral mass (100×), Sirius red pH 10.2.

It corresponded to multiple sphere-like or oval-shaped parenchymal granulomas adjacent to terminal bronchioles, sometimes isolated, but becoming confluent with the course of infection (**Figure 2A**). Nodules presented the following frequencies: 10±15, 34±26, 6±5 and 7±7 at 4, 8, 12 and 16 weeks, respectively (*p<0.05* at 8 weeks in reference to the other times). The mean diameter of the nodules was 360.2±SD 103.7 µm.

Their cellular composition varied according to time of infection. At 4 weeks, the granulomas were composed by a central core of macrophages with fungi and intermixed neutrophils sometimes presenting an apoptotic aspect (**Figure 2B**). Some macrophages formed multinucleated giant cells most of the foreign body type.

The most peripheral zone often consisted of non-continuous halo of small lymphocytes, at times forming pseudo-follicular lymphocyte aggregates.

At 8 weeks post-infection (p.i.), the nodules showed an increase in their number and a higher confluence among them. The most significant change in the central zone was the appearance of some xantomatous cells and cholesterol crystals. Additionally, the number of neutrophils significantly decreased and a large number of mature plasma cells appeared at the granuloma periphery.

At 12 weeks p.i., the xantomatous aspect of the macrophages and the number of cholesterol crystals were intensified (**Figures 2C, 2D**). Some of the granulomas were confluent and/or in direct contact with the periarterial lesions.

At 16 weeks p.i., nodules presented a significant increase in the number of xantomatous and Touton cells.

#### Inflammatory periarterial sheath pattern

At 4 weeks, the periarterial spaces were globally preserved but presented lymphatic dilatation and edema. The inflammatory cells consisted in only focal accumulation of small lymphocytes and some macrophages with neutral glycoproteins (PAS positive stain).

At 8 weeks, the involvement of this pulmonary compartment was more diffuse and defined periarterial inflammatory sheaths (**Figures 2E, 2F**). The inflammatory infiltrate consisted of a large number of macrophages, fungi, mature plasmocytes, small lymphocytes and eosinophils. The lymphatic vessels were more prominent.

The same aspects were also seen at 12 weeks p.i. except for presenting a higher number of eosinophils, plasmocytes and Mott cells (**Figures 2G, 2H**). At 16 weeks p.i., there was a reduction in the periarterial involvement.

Peri-venous inflammation occurred less frequently than arterial involvement with the former being more prominent at 12 weeks p.i. The perivenular cellular composition was similar to that in arteries.

#### Pseudotumoral pattern

Pseudotumoral masses were 5 to 6 times larger (2000±194 µm of length) than the nodules (**Figures 2I, 2J**). This pattern appeared mainly at 16 weeks p.i. and was the main lesion in 80% of mice at this time. Masses were adjacent to hilar bronchi and their large size produced bronchial compression (**Figure 2K**). In one case, the mass projected into a large bronchus, forming an endobronchial polyp (**Figure 2J**). These masses exhibited a combination of the main features described in the two previous patterns, and presented also a larger number of plasmocytes with plasmacytic differentiation (plasmocytogenesis) (**Figure 2L**) and eosinophils in the peripheral zone.

### Extracellular matrix aspects of the lesions

The aspect and quantity of extracellular matrix were dependent on the type of lesion and time of infection. Collagen fibers gradually increased up to 12 weeks, with further decrease at 16 weeks.

**Figure 3:**
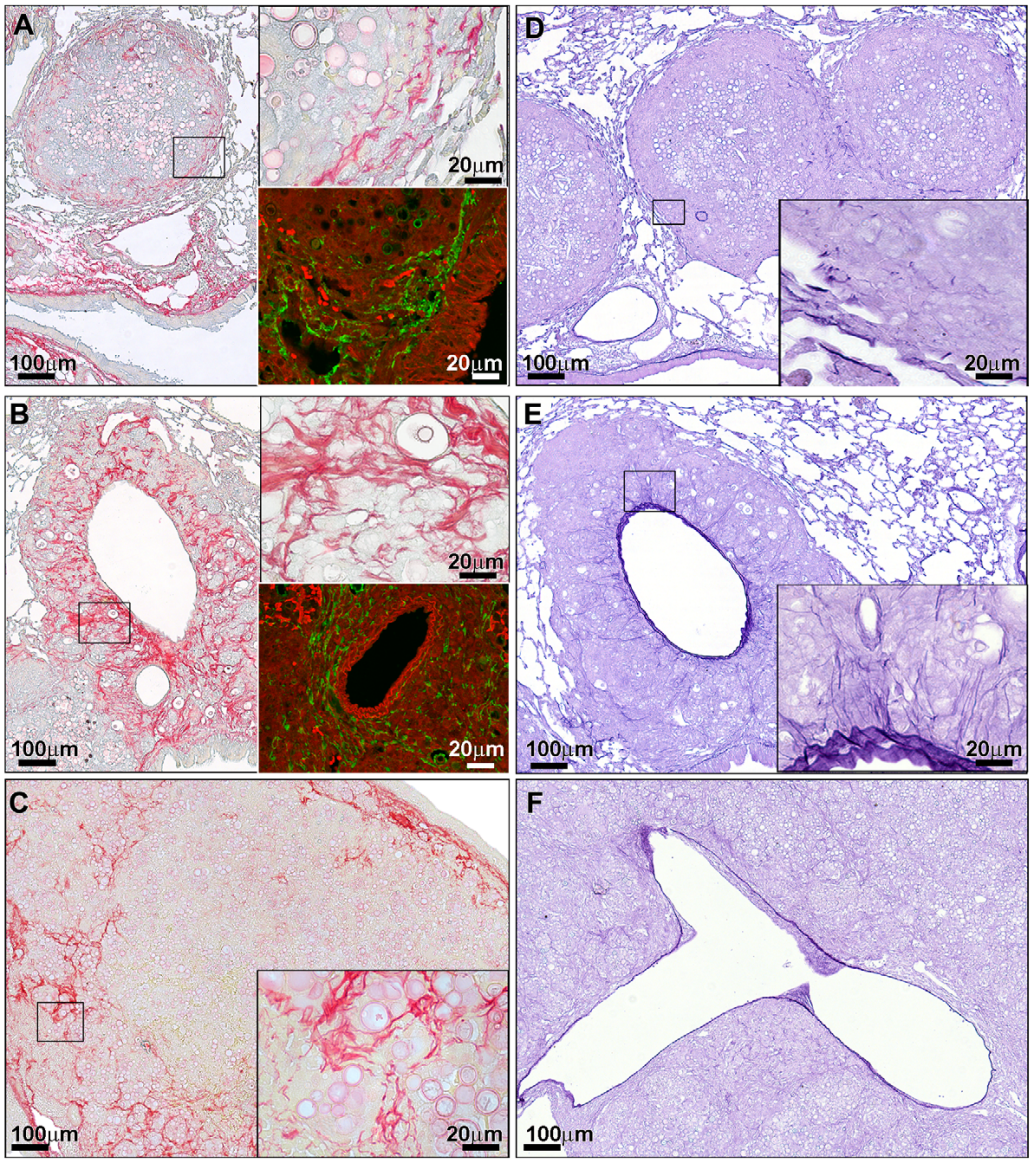
Extracellular matrix aspects observed in pulmonary lesions of experimental PCM. (A to C) PMA-picrosirius stain for collagen. (D to F) Weigert's resorcin-fuchsin stain for elastic fibers. Lower insert in A and B shows fibronectin expression observed by confocal indirect Immunofluorescence. (A) Nodule presenting a thin and concentric pseudo-capsule (10×), better exemplified in upper insert (100×). Lower insert in (A) shows fibronectin expression. (B) Periarterial space showing more abundant and thicker collagen fibers than the ones surrounding nodules (10×), detailed in the upper insert (100×). Lower insert in B shows a slight and diffuse fibronectin expression. (C) Pseudotumoral mass illustrating interstitial collagens with slight intensification at the periphery and around small cocoons (10×) (insert 100×). A large central area is devoid of collagen fibers. (D) Three adjacent nodules with intense elastolysis (10×). Insert shows tiny residual elastic fibers in the periphery of nodule (100×). (E) Periarterial space with radial elastogenesis (10×), detailed in the insert (100×). (F) Focal intimal vein thickening (10×).

Comparative features between extracellular matrix arrangements in the lesions are presented in the **Figure 3**.

### Fungal morphology

The morphology and quantity of the fungi in the lung tissue also varied depending on the location of the lesions and the time of the infection with a tendency to increase fungal numbers at 8 and 12 weeks post-infection.

In nodules, fungal cells were restricted to the central zone with predominance of large yeasts (10–20 µm). In comparison with the nodular pattern, fungi in perivascular lesions were more dispersed and frequently presenting a perifungal space surrounding a large mother cell with multiple small oval buds, less than 2 µm. These buds formed concentric aggregates around the mother cell and/or were dispersed in or attached to the external border of the perifungal space. Fungal cells were present in high amounts in pseudotumoral masses.

A summary of the main histopathologic aspects for each pulmonary lesion is represented in **Table 1**.

**Table 1:** Comparative overview of the main characteristics of each pulmonary lesion pattern in experimental model of PCM.

	Nodular	Peri-arterial sheath	Pseudotumoral masses
Location	Parenchyma, adjacent to terminal bronchiole	Around arteries	Upper third of lung, mainly hilar
Shape	Sphere-like or oval-shaped	Sheath-like	Sphere-like or oval-shaped
Size	360.2±SD 103.7 µm in diameter/length	Variable	2000±194 µm of length
Time of maximum appearance	8th week post-infection	12th week post-infection	16th week post-infection
Plasmocytes	+/++	+++	+++
Eosinophils	+	+++	+++
Yeasts with perifungal space	+	+++	+
Interstitial Collagen	+	+++	++
Elastic fibers	Elastolysis	Elastogenesis	Elastolysis

### Immunological aspects

**Figure 4:**
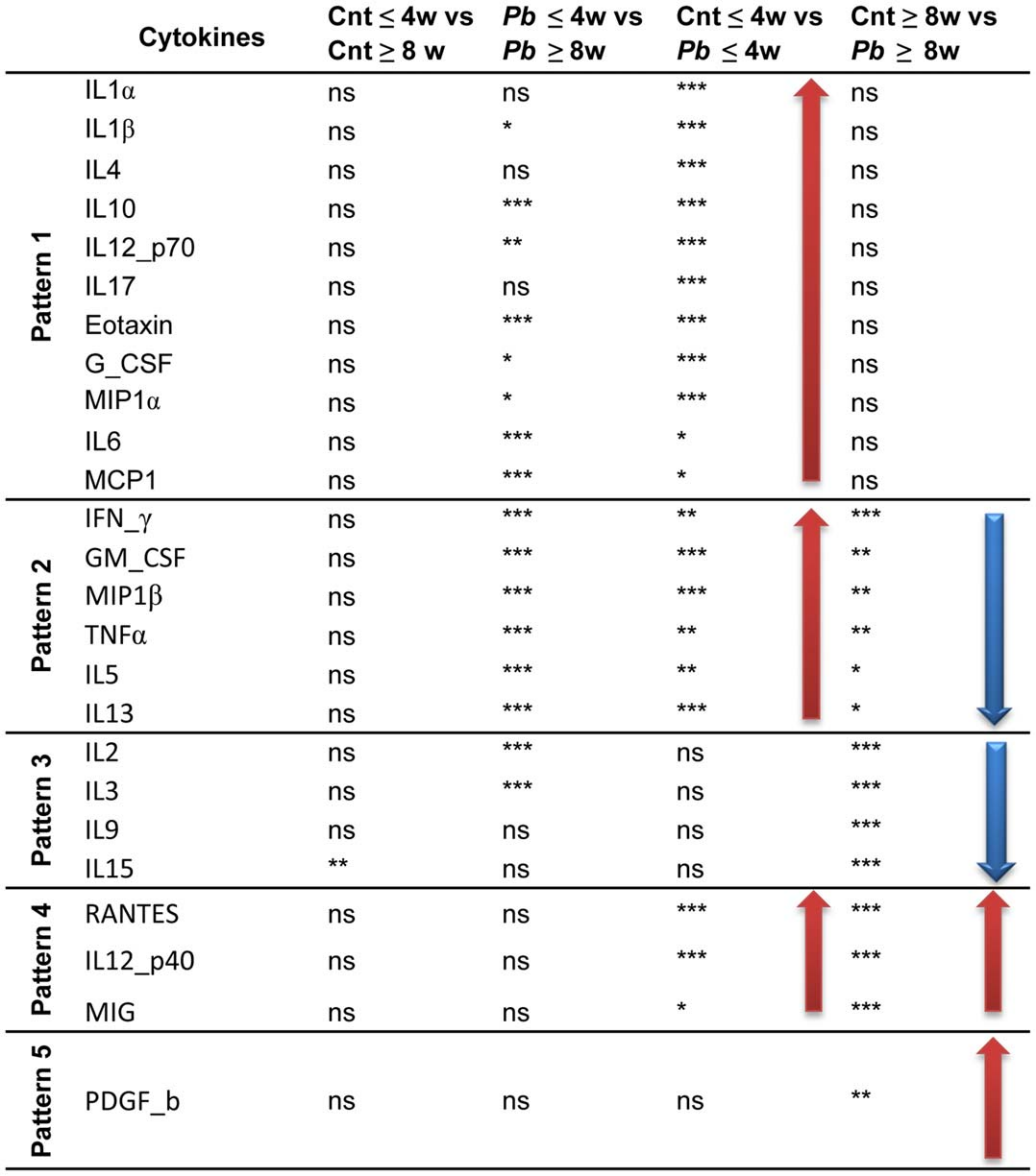
Pulmonary cytokine level comparisons between *Pb*-infected and control mouse groups. Control and *Pb*-infected groups were divided in two sub-groups according to time: up to 4 weeks and after 8 weeks. Subgroups were compared by Kruskal-Wallis one-way ANOVA test followed by Dunn's multiple comparison post test, at the 0.05 significance level. ns = non-significant * = *p≤0.05*, ** = p≤0.01, *** = p≤0.001.

By means of exploratory data analysis, the kinetic of each cytokine was plotted against time of infection using Lowess smoothing (40%). The observed trajectories (data not showed) clearly showed a switch of some cytokine levels up to 4- and from 8- weeks afterwards. To test this statistical hypothesis, each cytokine in the *Pb*-infected group was compared with those of the control group by the Kruskal-Wallis test using two time periods consisting of two hours up to four weeks and eight to sixteen weeks of infection (**Figure 4**). These analyses enabled us to identify five different patterns of inflammatory mediators, as follows: (a) Pattern 1: molecules significantly up regulated until the 4 week and then becoming normalized (IL-1α, IL-1β, IL-4, IL-6, IL-10, IL-12p70, IL-17, Eotaxin, G-CSF, MCP1 and MIP1α); (b) Pattern 2: molecules significantly up regulated up to 4 weeks and then significantly down regulated (IL-5, IL-13, GM-CSF, IFN-γ, MIP1β and TNFα); (c) Pattern 3: molecules without significant changes up to 4 weeks but significantly down-regulated after 8 weeks p.i. (IL-2, IL-3, IL-9, IL-15); (d) Pattern 4: molecules significantly up-regulated at all times p.i. (MIG, RANTES, and IL-12p40); and (e) Pattern 5: composed only by one molecule (PDGF) without significant changes up to 4 weeks but significantly up-regulated after 8 weeks p.i. In the control group, cytokine levels showed no significant variation with time except for IL15 that increased with observation period.

## Discussion

The overall results of this study, once integrated, have allowed to differentiate five chronological stages occurring during the course of experimental PCM induced by inhalation of infective *P.b* conidia, as follows: In the first stage (2 hours p.i.), a mild septal neutrophilic and macrophagic infiltration accompanied by an intense “cytokine burst” was observed in lung tissues as shown by a significant increase of IL-1α, IL-1β, IL-4, IL-5, IL-6, IL-10, IL-12p70, IL-13, IL-17, Eotaxin, G-CSF, MCP1, MIP1α, GM-CSF, IFN-γ, MIP1β and TNFα (patterns 1 and 2).

In the second stage (4 wks p.i.), nodules and incipient periarterial inflammatory reaction, together with disperse and intense parenchymal inflammation became evident. In addition, hyper-secretion of the cytokines and chemokines grouped in patterns 1 and 2 was also recorded.

In the third and fourth stages (8 and 12 wks p.i., respectively) fungal growth, inflammation and collagenesis reached their highest intensity with particular involvement of the periarterial space. Paradoxically at this point, lung cytokines and chemokines were down-regulated as revealed by significant decreases in IL-5, IL-13, GM-CSF, IFN-γ, MIP1β, TNFα, IL-2, IL-3, IL-9 and IL-15 (patterns 2 and 3).

Finally, in the fifth stage (16 wks p.i.), the inflammation decreased and became practically restricted to the pseudotumoral masses which were now accompanied by a “silent” cytokine response, except for PDGF, and MIG, RANTES and IL12p40, all of which remained up- regulated during the whole experimental period.

Some associations among morphological changes and cytokine profiles could be attempted, as follows:

- High levels of IL-1α, IL-1β, IL-6, IL-17, TNFα and chemokines such as eotaxin, MCP1 and MIP1α indicated a highly proinflammatory milieu at 2 h and 4 wks p.i. and would explain the recruitment of monocytes, PMNs and eosinophils [26].
- During the first 4 wks p.i, high levels of both Th2 cytokines (IL-4, IL-5, IL-13) and Th1 cytokines (IFN-γ, IL-12p70) were detected, indicating that during this particular infectious stage no polarization of these molecules had occurred in the lungs of *Pb*-infected mice. Nonetheless, IL-4 remained up-regulated for another 4 wks p.i. (data not shown) to diminish later on until reaching normal levels. Expression of IL-4, IL-5 and IL-13 are associated with airway hypersensitivity, eosinophilia, abundant plasma cells and pathological changes in the lungs [27].
- IFN-γ is considered to mediate resistance to PCM [28], and in this experiments high levels of this cytokine were observed only at 2 h and 4 wks p.i. at the same time with occurrence of the pro-inflammatory burst. Later on, however, IFN-γ decreased to lower levels than those in controls. This change towards IFN-γ down-regulation together with low IL-2 levels has been also regularly observed in patients with chronic PCM [29].
- For the first 4 wks p.i., pro-inflammatory cytokines, chemokines and growth factors co-existed with high concentrations of immune-regulators such as IL-10 and IL-12p40. IL-10 hinder full activation of monocytes/macrophages and T cells decreasing their fungicidal activity [30]. IL-12p40 is a competitive antagonist of IL-12p70 and also acts as a chemoattractant for macrophages [31]. As pro and anti-inflammatory stimuli co-existed, it was not possible to determine the predominant cytokine effect.
- MIG (CXCL9), RANTES (CCL5) and IL-12p40 were up-regulated at all times during the experimental period, including most chronic times (8, 12 and 16 wks) when the remaining cytokines decreased. The molecules referred to above could be considered as markers when discriminating PCM cases from controls and could also be useful when treatment follow-up is considered. Indeed, in a study with human PCM patients, serum levels of MIG/CXCL9 were higher in all untreated cases and decreased progressively with treatment [32]. In addition, MIG, RANTES and IL12p40 appeared to be of help in maintaining cellular recruitment during the chronic periods of the *Pb*-infection when the remaining chemokines showed low levels. MIG is a chemotactic factor selective for lymphocytes which exerts its greatest influence on activated CD4+ Th1 cells, CD8+ T cells and NK cells [33]. RANTES is a potent chemokine for monocytes, macrophages, T cells, dendritic cells, eosinophils, and basophils [34]. IL12p40 also acts as a chemokine for macrophages [31].

In the course of these studies we have noticed certain histopathological findings that due to their novelty deserve further exploration, as discussed below:

- Foamy cells were frequent in the granuloma central areas, as were Touton cells and large cholesterol crystals suggesting participation of lipids in *Pb*-infection. Foamy macrophages have been described in individuals developing secondary or adult type tuberculosis [35], in *Mycobacterium avium*-infected AIDS patients, in chronic stages of *M. tuberculosis* infection in mice [36], [37] and in *Chlamydia* infections [38]. However, their presence or significance has not been often discussed in PCM.
- Another peculiar feature in experimental PCM was the involvement of the periaterial space. Although this compartment has been regularly neglected, its involvement has been reported in several animal models, such as respiratory syncycial virus, bacteria including *M. tuberculosis*, in some asthma models and in lipopolysaccharide-induced inflammation [39]. The mechanisms and route of recruitment of inflammatory cells into the perivascular space (PVS) remain largely unknown. An important feature in the peri-arterial sheath inflammation was the higher frequency of plasmocytes and eosinophils in comparison to those existing in nodules and granulomas. These cells are associated with a Th2 cytokine profile, as well as with antibody production [40], [41]. The participation of B-lymphocytes in paracoccidioidomycosis has not been completely elucidated; however, B-1 cells contribution to susceptibility in experimental disease has been reported [42], [43]. In addition, activation of B- cells is associated with a severe disease outcome and is indicative of a progressive disease [44]. Mott cells containing Russell bodies indicate plasma cells indigestion due to a failure to eliminate misfolded or incorrectly assembled proteins [45], [46].
- We reported pseudotumoral masses in the lungs of *Pb*-infected mice. Pulmonary masses have been recorded in patients in whom chest x-rays revealed dense large solitary masses with well-defined borders sometimes referred as paracoccidioidomas [47]–[49]. The preference of this lesion for the hilar zone could probably be explained by the characteristics of the pulmonary lymph flow which proceeds in opposite directions: lymphatics in the lung perimeter flow to the pleura and the rest flows towards the hilum [50].

These overall results complement previous work by our group [51], where noninvasive conventional medical X-ray tomography allowed the *in vivo* follow-up of the three main pathological patterns described above.

In conclusion, this study has revealed different patterns of pulmonary lesions according to time post-infection, as well as different cytokine trajectories both of which have allowed the description of five stages in the development of experimental PCM. Recognizing those stages in the course of the mycosis could help us to predict the severity (benign, chronic or fibrotic) of this disease and infer the probable prognosis of the illness.

Finally, this study has open up future possibilities to investigate the mechanisms involved in the different morphological patterns described, as well as gaining new insights into the pathogenesis of PCM.
